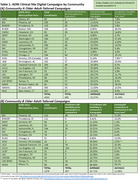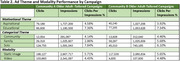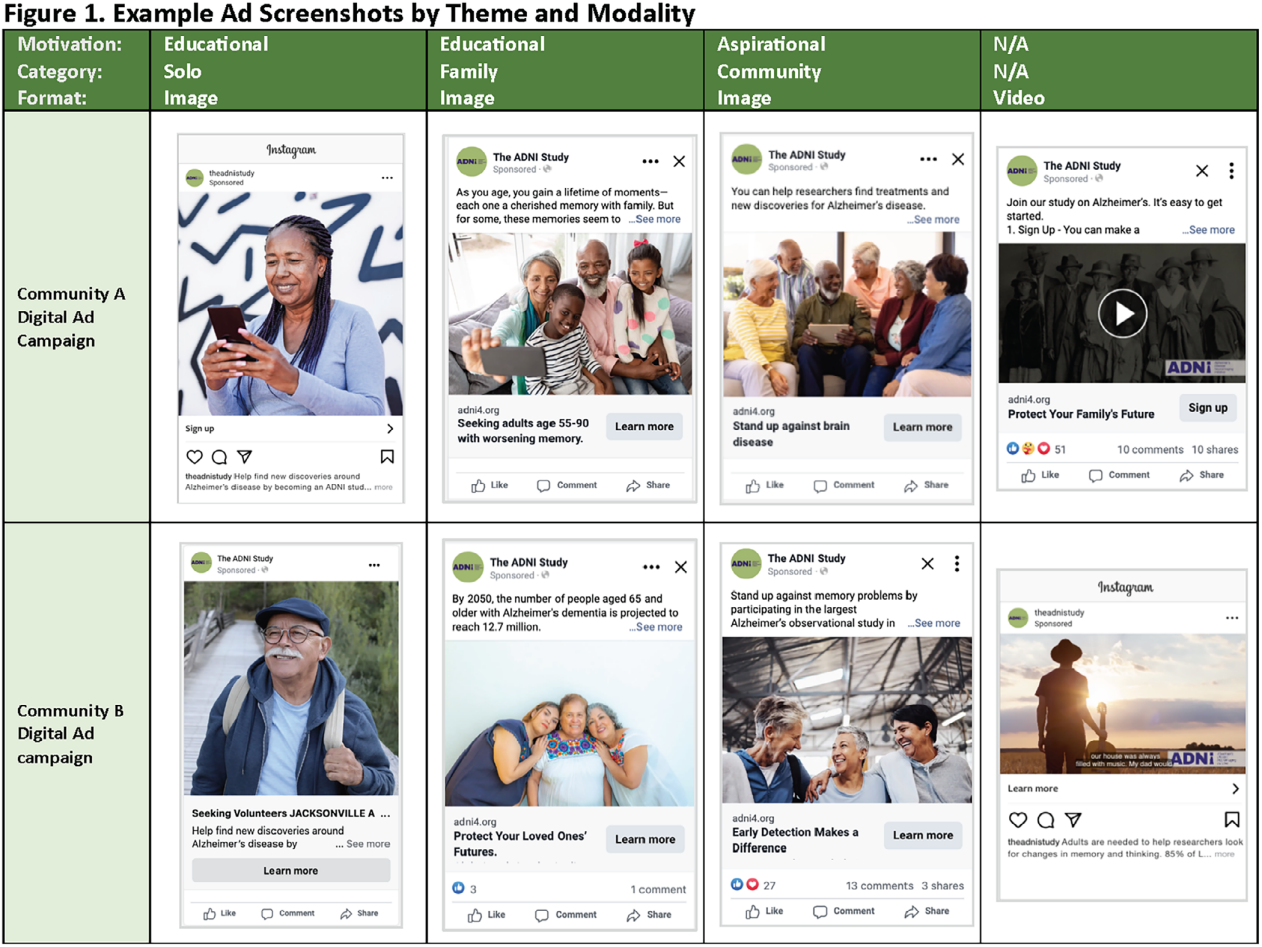# Demographic Community‐Focused Digital Advertising for Recruitment into the ADNI4 Digital Study

**DOI:** 10.1002/alz70860_099539

**Published:** 2025-12-23

**Authors:** Catherine C. Conti, Hannatu Amaza, Melanie J. Miller, Adam Diaz, Mai Seng Thao, Derek Flenniken, Winnie Kwang, Miriam T. Ashford, Juliet Fockler, Diana Truran‐Sacrey, Jennefer Sorce, Roxanne Alaniz, Michael S. W. Weiner, Ozioma C. Okonkwo, Monica G Rivera Mindt, Rachel L. Nosheny

**Affiliations:** ^1^ Northern California Institute for Research and Education (NCIRE), San Francisco, CA, USA; ^2^ Wisconsin Alzheimer's Disease Research Center, School of Medicine and Public Health, University of Wisconsin‐Madison, Madison, WI, USA; ^3^ University of California, San Francisco, San Francisco, CA, USA; ^4^ Alaniz Marketing, Novato, CA, USA; ^5^ Icahn School of Medicine, Mount Sinai Hospital, New York, NY, USA; ^6^ Fordham University, New York, NY, USA

## Abstract

**Background:**

The Alzheimer's Disease Neuroimaging Initiative (ADNI4) aims to increase participation of individuals from different communities so results are more generalizable. ADNI uses digital marketing to recruit and enroll older adults into a Digital cohort for screening into In‐Clinic. This abstract presents ad engagement data by ad theme and modality, and enrollment data.

**Method:**

We employed a demographic community‐focused marketing approach developed in partnership with ADNI's Community Scientific Partnership Board and marketing firm. Digital ads were run in geographic areas near ADNI sites based on site readiness to enroll participants and informed by the older adult population's demographic composition in those areas, estimated using American Community Survey (ACS) data for adults age 65+. Digital campaigns (Meta ads, landing pages, emails) used themes (solo, family, community; educational content, aspirational messaging) designed to resonate with different communities in static image or video format (Figure 1). Varying ads were run simultaneously to compare effectiveness. Ads directed potential participants to study websites for Digital cohort enrollment. Select Digital participants were referred for site screening into the In‐Clinic cohort. Ad engagement was estimated as the ratio of clicks (clicks on ad) to impressions (ad views).

**Result:**

Digital campaigns enrolled 5,931 participants between 9/18/2023 and 1/21/2025 into the Digital cohort. Most campaigns enrolled the desired community group at a higher percentage than the associated metropolitan area's population. Overall, we enrolled 22% from one community and 32% from another, compared to ACS estimates of 13.39% and 11.09% (Table 1), helping improve generalizability of our study. Ad themes and format types performed similarly across different community campaigns. In both, “solo” messaging‐themed ads yielded highest engagement. Ads with educational content yielded higher engagement compared to aspirational ads; and a higher percentage of those shown image ads clicked than those shown video (Table 2). ADNI sites screened 18 participants traced from ad campaigns into the In‐Clinic cohort.

**Conclusion:**

Successful recruitment of adults from different communities demonstrates the feasibility of community‐focused, site‐specific digital marketing for the ADNI Digital study cohort. High engagement with ads of solo themes, educational content, and static imagery demonstrates these ad configurations are most effective for both communities.